# Supplementary material for: Aetiologies and Risk Factors of Prolonged Fever Admission in Samtse Hospital, Bhutan, 2020
Source: Int J Environ Res Public Health. 2022 Jun 27;19(13):7859. doi: 10.3390/ijerph19137859 (PMC9266161; doi:10.3390/ijerph19137859)
Supplement: Supplementary file 1 [file ijerph-19-07859-s001.zip › ijerph-1755295-supplementary.pdf]

Supplementary Tables

Supplementary Table S1. Pro forma used to collect data for fever admissions in Samtse hospital, Bhutan, 2020.

<u>**Variables**</u>	**Data**	**Responses**
Registration no	Admission registration no	
Age (years)	Age in years	
Age (mths)	Age in months	
Sex	Gender	Male<br>Female
Occupation	Occupation	Farmer<br>Housewife<br>Businessman/woman<br>Driver<br>Corporate worker<br>Dependent<br>Civil servant<br>Armed forces
Add-gewog	Address-gewog	
Add-dist	Address-district	
Date of admn	Date of admission	
Referred/OPD	Referred/OPD case	Referred in<br>Direct walk in
Fever (degrees C)	Temperature in ° C	
Symptoms (multiple responses allowed)	Symptoms (multiple responses allowed)	Cough<br>Shortness of breath<br>Others
Other symptoms	Other symptoms	Other associated symptoms
Co-morbidities (multiple responses allowed)	Co-morbid conditions (multiple responses allowed)	DM<br>HTN<br>COPD<br>ALD<br>Anaemia

		Heart failure<br>Cancer<br>CKD<br>Tuberculosis
Diagnosis	Diagnosis	Diagnosis on Discharge
Antimicrobial agent (multiple responses allowed)	Antibiotics and antivirals received (multiple responses allowed)	Ceftriaxone<br>Ampicillin<br>Ciprofloxacin<br>Metronidazole<br>Amoxicillin<br>Cloxacillin<br>Gentamicin<br>Doxycycline<br>Oseltamivir
Other treatments	Other treatments	Other supportive or symptomatic treatment
Duration of admn	Duration of admission	Date of admission<br>Date of discharge
Outcomes	Outcomes of illness	Discharged<br>Referred<br>Expired

Supplementary Table S2. Median length of stay categorized according to demographic characteristics of patients admitted with febrile illness in Samtse hospital, Bhutan, 2020.

Variables	Category	Median	Interquartile range	p value
Age groups (years)				
	<12	4	4 (3–7)	0.776
	>12	4	3 (3–6)	
Sex				
	Male	4	4 (3–7)	0.609
	Female	4	3 (3–6)	
Occupation				
	Farmer	4	4 (3–7)	0.496
	Housewife	4	4 (3–7)	
	Children/student	4	3 (3–6)	
	Others	4	2 (3–5)	
Residence				
	Urban	3	2 (3–5)	0.002
	Rural	4	4 (3–7)	
Source				
	Catchment population of Samtse Hospital	4	3 (3–6)	0.214
	Referred	5	5 (3–8)	

Supplementary Table S3. Comparison between urban and rural patients with co-morbidities among fever admissions in Samtse hospital, Bhutan, 2020

Co-morbidities	Total	Urban	Rural	p value
Hypertension	12 (21.4)	2 (50.0)	10 (19.2)	0.025
Diabetes	7 (12.5)	2 (50.0)	5 (9.6)	
Alcohol liver disease	7 (12.5)	0 (0.0)	7 (13.5)	
*Others	30 (53.6)	0 (0.0)	30 (57.7)	

*Others: anaemia, malnutrition, seizure, stroke, sexually transmitted infections, heart and kidney disease.
